# Transcriptomic effects of di-(2-ethylhexyl)-phthalate in Syrian hamster embryo cells: an important role of early cytoskeleton disturbances in carcinogenesis?

**DOI:** 10.1186/1471-2164-12-524

**Published:** 2011-10-25

**Authors:** Yann Landkocz, Pascal Poupin, Franck Atienzar, Paule Vasseur

**Affiliations:** 1 CNRS UMR7146, Laboratoire I.E.B.E., Rue General Delestraint, 57070 Metz, France; 2 UCB Pharma SA, Non-Clinical Development, Chemin du Foriest, 1420 Braine-l'Alleud, Belgium

## Abstract

**Background:**

Di-(2-ethylhexyl)-phthalate (DEHP) is a commonly used plasticizer in polyvinylchloride (PVC) formulations and a potentially non-genotoxic carcinogen. The aim of this study was to identify genes whose level of expression is altered by DEHP by using a global wide-genome approach in Syrian hamster embryo (SHE) cells, a model similar to human cells regarding their responses to this type of carcinogen. With mRNA Differential Display (DD), we analysed the transcriptional regulation of SHE cells exposed to 0, 12.5, 25 and 50 μM of DEHP for 24 hrs, conditions which induced neoplastic transformation of these cells. A real-time quantitative polymerase chain reaction (qPCR) was used to confirm differential expression of genes identified by DD.

**Results:**

Gene expression profiling showed 178 differentially-expressed fragments corresponding to 122 genes after tblastx comparisons, 79 up-regulated and 43 down-regulated. The genes of interest were involved in many biological pathways, including signal transduction, regulation of the cytoskeleton, xenobiotic metabolism, apoptosis, lipidogenesis, protein conformation, transport and cell cycle. We then focused particularly on genes involved in the regulation of the cytoskeleton, one of the processes occurring during carcinogenesis and in the early steps of neoplastic transformation. Twenty one cytoskeleton-related genes were studied by qPCR. The down-regulated genes were involved in focal adhesion or cell junction. The up-regulated genes were involved in the regulation of the actin cytoskeleton and this would suggest a role of cellular plasticity in the mechanism of chemical carcinogenesis. The gene expression changes identified in the present study were PPAR-independent.

**Conclusion:**

This study identified a set of genes whose expression is altered by DEHP exposure in mammalian embryo cells. This is the first study that elucidates the genomic changes of DEHP involved in the organization of the cytoskeleton. The latter genes may be candidates as biomarkers predictive of early events in the multistep carcinogenic process.

## Background

Di-(2-ethylhexyl)-phthalate (DEHP) is a commonly used plasticizer in polyvinylchloride (PVC) formulations which have a number of applications, especially in food packaging, medical devices or cosmetics. Phthalates are not chemically bound to PVC and can migrate from PVC-containing products to the environment, resulting in significant environmental contamination and human exposure [1,2].

DEHP experiments have revealed toxicities including carcinogenesis and endocrine-disrupting effects, but no genotoxicity has been recorded [3]. DEHP is capable of disturbing the reproductive process by mimicking or antagonizing steroid hormone action [4] and its effects on testosterone, luteinizing hormone or estrogen-like activity have been reported [5,6]. DEHP has been shown to decrease free testosterone levels in humans after occupational exposure [7] and thyroid hormone levels in adult men otherwise exposed [8].

DEHP has been classified as a peroxisomal proliferator and as a non-genotoxic carcinogen in animals [9]. Experimental studies using rodents and *in vitro *assays showed that DEHP and its active metabolite MEHP (mono-(2-ethylhexyl)-phthalate) can interact with nuclear receptors like PPARα [10] or PPARγ [11]. Oxidative stress, as a result of peroxisome proliferation, and DNA damage have been described in the human prostate adenocarcinoma cell line LNCaP [12,13] and the mouse Leydig tumor cell line MA-10 [14] exposed to high concentrations of DEHP (3 mM). Peroxisome proliferation is one of the mechanisms that produce liver tumors in rats or mice, but this mechanism was not judged to be relevant in humans [15]. The liver is not the sole target for DEHP carcinogenicity: testicular tumors [16] and pancreatic acinar adenomas have also been reported [17]. Other studies have pointed out that peroxisome proliferation is not a necessarily pathway in the carcinogenicity of DEHP [18] and more liver tumors occurred in PPARα-null mice than in wild type animals [19]. Transcriptional changes independent of PPARα were also found in rats and mice exposed to DEHP [20]. Several non-PPARα mechanisms were addressed: activation of p38 mitogen-activated protein kinase not involved in peroxisome proliferations [21]; stimulation of growth regulatory pathways, mitogen-activated protein kinase, extracellular signal-regulated kinase and p38 phosphorylation [22]. Other mechanisms related to non-genotoxic carcinogenicity, like inhibition of gap junctional intercellular communication [23] or inhibition of apoptosis, were reported. Apoptosis was shown to be suppressed by DEHP through different pathways. An interference with the cytokine TGF-β1 (transforming growth factor-β1) [24] or with TNF-α (tumor necrosis factor-α) has been described [25]. An increased level of Bcl-2 and negative regulation of c-Myc expression has been related to inhibition of apoptosis in Syrian hamster embryo cells treated with 50 μM of DEHP [26].

Several authors have demonstrated that DEHP and its active metabolite MEHP induce morphological transformation of SHE cells [27-29], indicating the carcinogenic potency of the two chemicals. Although phthalate toxicity has been extensively investigated over the past 10 years, the mechanisms of DEHP carcinogenicity have not been elucidated. It was recently stated by the International Agency for Research on Cancer (IARC) that PPAR-independent mechanisms of DEHP carcinogenesis are necessary to be studied [http://monographs.iarc.fr/ENG/Publications/techrep42/TR42-18.pdf].

The choice of cellular models and methodologies is critical to the study of the phenomenon of carcinogenesis. Syrian hamster embryo cells are a relevant model for mechanistic studies of chemical carcinogenicity. SHE cells, unlike mouse and rat cells, are less responsive to peroxisomal proliferation and, in this respect, more similar to human cells. SHE cells are normal, diploid, genetically stable and primary cells which are metabolically competent for procarcinogen activation. Therefore they are used to study mechanisms of *in vitro *carcinogenesis [30]. SHE cells are obtained from embryos after removal of the differentiated tissues, and the population is mainly composed of epithelial and fibroblastic cells [31]. SHE cells from colonies having been morphologically transformed after short exposure to chemical carcinogens induced tumours when transplanted back into hamsters [32]. This validated the model and the cell transformation criteria for *in vitro *carcinogenicity. Recently, the SHE cell transformation assay has been recommended by OECD in 2007 as *in vitro *method of screening chemical carcinogens on the basis of its performances to detect non-genotoxic as well as genotoxic carcinogens [http://www.oecd.org/dataoecd/56/5/37863750.pdf].

The aim of this work was to use a global transcriptomic approach to understand the molecular mechanisms of cell transformation induced by DEHP in SHE cells. The objectives were to identify changes in gene expression occurring in the early steps of cell transformation as well as pathway disturbances that may trigger a carcinogenic process. A characterization of the genes expressed in SHE cells at DEHP concentrations inducing cell transformation may give information on PPAR-independent mechanisms and alternative pathways of DEHP carcinogenicity. The transcriptomic changes induced by DEHP in SHE cells were analyzed in the first hours of exposure. We focused secondly on changes of cytoskeleton-related genes underlying morphological transformation in SHE cells. Indeed, cell transformation is expressed by the alteration of cell morphology, a disorganized pattern of colony growth and the acquisition of anchorage-independent growth which is predictive of their ability to induce tumors when injected into syngenic animals [33]. Despite the central role of the actin cytoskeleton throughout the life cycle, little is known about the gene expression changes involved in deregulation of its dynamic in the first stages of tumorigenesis. Cytoskeleton defects in relation to cancer have been mostly studied in the late stages of cell invasion and metastasis.

Differential Display was chosen to identify differentially-expressed genes in SHE cells and to explore the entire genome. The mRNA differential display described by Liang and Pardee [34] is a powerful approach for transcriptomic analysis. This methodology has become popular as a tool for non-model organisms because of lack of requirement of previous genomic information about the species of interest. As the genome of hamster is partly characterized so far, Differential Display appeared quite appropriate to study DEHP dose-dependent effects in SHE cells. We applied the current methodology that uses a combination of 3 "anchored" oligo-dT primers (to divide the cDNA population in 3 subsets) and 80 "arbitrary" primers of 13-mers. The 240 primer combination allowed us to obtain a level of 95% gene coverage [35].

DD was applied to cells exposed for 24 hrs to DEHP. Genes corresponding to differentially-expressed fragments were characterized. Differential Display allowed us to screen a set of differentially-expressed fragments in treated cells, among which 122 genes were identified as targeted by a 24 hr-DEHP exposure. These genes were involved in such functions as transcription signalling pathways, cytoskeleton regulation, apoptosis, metabolism. As Differential Display is a semi-quantitative method, the expression changes of the genes we were interested in, were checked by qPCR using hamster specific primers. qPCR was applied to RNAs not only from 24 hr-treated cells, but also from cells treated for 5 hrs in order to study the cell response in the meantime. We particularly focused on changes of cytoskeleton-related genes underlying morphological transformation in SHE cells. The objective was to explain from a mechanistic point of view the gene expression changes after DEHP exposure. To the best of our knowledge, this exercise has never been done previously.

## Results

### Identification of DEHP-responsive genes using Differential Display

**Figure 1:**
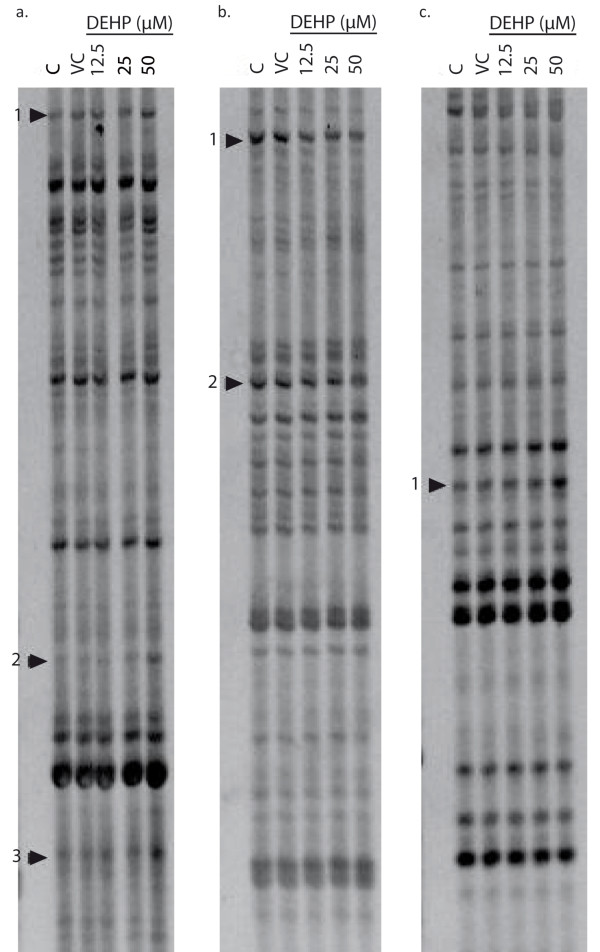
**Representative Differential Display**. This figure shows results obtained with control (C), vehicle control (VC) and DEHP-treated (12.5, 25 and 50 μM) SHE cells mRNA, after 24 hrs of exposure. Arrows indicate fragments regulated more than 2-fold and used for the next step of analysis. The DD fragments were generated using the A-anchored primer (H-dT+A) combined with the arbitrary primer H-AP37 (lane a) or H-AP52 (lane b) and the G-anchored primer (H-dt+G) combined with the arbitrary primer H-AP40 (lane c). After characterization by sequencing, we obtained the following genes: a1:Rlp37a/a2:Rlp27a/a3:Col1a1/b1:Cttnbip1/b2: Enah/c1: Coro1C.

The Differential Display technique was used to identify genes differentially expressed in SHE cells, after 24 hrs of treatment with DEHP. An illustration of differentially-expressed fragments is given in Figure 1 which shows gels obtained after the DD protocol and highlights fragments regulated more than 2-fold by DEHP. Using 3 anchored primers and 80 arbitrary primers, 178 differentially expressed fragments were identified (115 up-regulated and 63 down-regulated). Among these transcripts, 141 (79%) showed homology to known genes in the RefSeq database (mouse or human) of Genbank, while 37 (21%) had no homology or homology to hypothetical proteins. The sequences of the fragments obtained by DD have been deposited in the Genbank dbEST database (LIBEST_027390 Syrian Hamster Embryo cells library). These 141 fragments corresponded to 122 genes that are listed in table 1 with their accession numbers and the tblastx expected (the threshold for tblastx comparison was p ≤ 0.001). These genes were classed according to 8 biological functions with reference to the GO process database. These functions included signal transduction and transcription, cytoskeleton regulation, xenobiotic metabolism, apoptosis, lipidogenesis, protein conformation or transport and cell cycle. The regulation of the cytoskeleton was one of the most impacted pathways. Indeed, 21 genes involved in this function were differentially expressed after DEHP exposure. Ten genes were up-regulated, and 11 were down-regulated.

**Table 1:** List of genes identified by Differential Display after DEHP exposure and classified by major biological function, according to GO process

Id	Official symbol	Gene Name	Accession Nr	Tblastx expect	Effect of DEHP
**1-Transcription and Signal Transduction**					
**A0601**	Hsp90	Heat shock protein 90 kDa protein	NM_010478	9E-25	+
**A0602**	Hsp70	Heat shock protein 70 kDa protein	NM_005346	1E-24	+
**A3201**	Cebpd	CCAAT/enhancer binding protein delta	NM_005195	9E-10	-
**A4102**	Ncbp2	nuclear cap binding protein subunit 2	NM_007362	6E-21	+
**A5101**	Mettl5	methyltransferase like 5	NM_029280	7E-10	+
**A5102**	Ogt	O-linked N-acetylglucosamine transferase	NM_181672	5E-28	+
**A6101** **A6102**	Nr1i2	nuclear receptor subfamily 1I2	NM_003889	1E-158 3E-74	+
**A6302**	Mapk15	mitogen-activated protein kinase 15	NM_177922	3E-16	+
**A6801**	Rab1b	member of RAS oncogene family	NM_030981	1E-22	++
**A7502**	Mdb1	methyl-CpG binding domain protein 1	NM_013594	2E-16	--
**C0602**	Gper	G protein-coupled estrogen receptor 1	NM_001098201	6E-68	+
**C1001**	Hmbox1	Homeobox 1	NM_024567	2E-32	++
**C1002**	Mapk3	mitogen-activated protein kinase 3	NM_001109891	9E-60	-
**C1301**	Usp3	Ubiquitin peptidase 3	NM_006537	9E-34	+
**C1401**	Psmc5	26S protease regulatory subunit ATPase 5	NM_002805	2E-23	-
**C4601**	Hsph1	heat shock 105kDa/110kDa protein	NM_006644	5E-07	+
**C4802**	Irf2	interferon regulatory factor 2	NM_008391	8E-19	+
**C7101**	Lmo4	LIM domain only 4	NM_006769	7E-09	+
**G0301**	Smarcc1	SWI/SNF related actin dependent regulator of chromatin	NM_003074	3E-20	-
**G1001**	Chd4	ATP dependant hélicase 4	NM_001273	1E-45	-
**G1504**	Foxp1	Forkhead box P1	NM_032682	3E-50	++
**G1701**	Creb3l1	cAMP responsive element binding protein 3-like 1	NM_052854	8E-19	+
**G1902**	Pbrm	Polybromo domain	NM_001081251	8E-49	+
**G3301**	Rxfp2	relaxin/insulin-like family peptide receptor 2	NM_130806	1E-24	+
**G3501**	Akap5	A kinase anchor protein 5	NM_004857	1E-05	+
**G3801** **G7001**	Eif1	eukaryotic translation initiation factor 1	NM_005801	3E-42 1E-15	+
**G4801**	Spry3	sprouty homolog 3	NM_005840	0.0002	+
**G6001**	Foxa3	forkhead box A3	NM_008260	9E-17	-
**G6201**	Mett5d1	methyltransferase 5 containing 1	NM_029790	0.0038	+
**2-Regulation of cytoskeleton**					
**A0201**	Cttnbp2	Cortactin binding protein 2	NM_030249	0.0001	+
**A1901**	Snx6	sorting nexin 6	NM_021249	8E-10	-
**A2401**	Lrrc8a	leucine rich repeat containing 8A	NM_177725	5E-32	-
**A3501** **A3502** **C3501** **G3502** **G3503**	Actb	beta-actin	NM_007393	3E-51 1E-106 2E-21 7E-114 3E-48	+
**A3702**	Col1a1	collagen, type I, alpha 1	NM_000088	1E-21	+
**A3801**	Nrp2	neuropilin 2	NM_0010774	1E-22	-
**A5201**	Ctnnbip1	catenin beta interacting protein 1	NM_020248	0.0008	-
**A5202**	Enah	enabled homolog	NM_008680	2E-09	-
**A5402**	Kif23	kinesin family member 23	NM_024245.4	1E-25	++
**A7501**	Cdh3	cadherin 3	NM_007665	0.0007	-
**C4502**	Nid2	nidogen 2	NM_008695	0.006	-
**C6603** **C6604** **C6605**	Crip1	cysteine-rich protein 1	NM_007763	1E-31 9E-08 3E-61	+
**G0601**	Thy1	Thy-1 cell surface antigen	NM_009382.3	0.001	-
**G0801**	Calml3	calmodulin-like 3	NM_027416.3	0.0004	+
**G1102**	Flrt2	fibronectin leucine rich transmembrane protein 2	NM_201518	2E-08	-
**G1301**	Has2	hyaluronan synthase 2	NM_008216	8E-18	-
**G1901**	Plekha5	Pleckstrin homology domain A5	NM_019012	3E-06	+
**G2301**	Thbs1	thrombospondin 1	NM_011580	3E-20	-
**G4001**	Coro1C	coronin, actin binding protein	NM_014325	4E-07	+
**G4802**	Tubb2b	Tubulin beta	NM_023716	0.0001	+
**G6901**	Dclk1	doublecortin-like kinase 1	NM_019978	1E-06	+
**3-Xenobiotic metabolism**					
**A0901**	Cyp2e1	Cytochrome P450 2e1	NM_021282	3E-44	+
**A2901**	Ephx1	Epoxide Hydrolase 1	NM_000120	4E-21	+
**A5701**	Gstp1	glutathione S-transferase, pi 1	NM_000852	1E-24	+
**A6601**	Gstt1	glutathione S-transferase, theta 1	NM_008185	1E-44	-
**C0101** **C1501** **G1502**	Txnrd1	Thioredoxin reductase 1	NM_001042523	1E-20 1E-14 3E-79	+
**C2901**	Cyp1b1	Cytochrome P450 1b1	NM_009994	0.0	+
**C3101**	Gstm5	glutathione S-transferase, mu 5	NM_010360	4E-51	-
**C3701**	Tnfa	tumor necrosis factor-alpha	NM_011659	0.00009	+
**G0101**	Psme4	proteasome activator subunit 4	NM_134013	4E-87	+
**G0701**	Mt2a	metallothionein 2A	NM_005953	7E-20	-
**G1201**	Ggt1	gamma-glutamyltransferase 1	NM_013430	3E-46	+
**G1501**	Txnrd2	Thioredoxin reductase 2	NM_006440	9E-19	+
**G2001**	Pdia4	Disulfite Isomerase 4	NM_004911	5E-46	+
**G2202**	Ahcy	S-adenosylhomocystein hydrolase	NM_016661	1E-10	-
**G2302**	Cyp2f2	Cytochrome P450 2f2	NM_007817	9E-25	--
**G3302**	Cytb	Mesocricetus auratus cytochrome b	YP_003208313	9E-86	++
**4-Apoptosis**					
**A4301**	Pik3r1	phosphatidylinositol 3-kinase	NM_001024955	1E-80	+
**C1701**	Tp53	tumor supressor p53	U07182	6E-83	-
**C7103** **G3701**	Bcl10	B-cell CLL/lymphoma 10	NM_003921	4E-37 5E-82	+
**G1503**	Nfkb1	NF-κB	NM_008689	2E-102	+
**G2201**	Casp8	Caspase 8	NM_009812	6E-29	-
**G2305**	Eef1d	Eukaryotic translation elongation factor 1 delta	NM_029663	3E-17	+
**G3001**	Topors	topoisomerase I p53-binding	NM_134097	4E-10	-
**G4101** **G4102**	Sh3kbp1	SH3-domain kinase binding protein 1	NM_031892	1E-09 0.0008	+
**G5301**	Cmyc	myelocytomatosis oncogene	AJ582076	2E-27	-
**5-Lipidogenesis**					
**A0501**	Pla2g2d	phospholipase A2, group IID	NM_011109	0.00003	+
**A3003**	Dhcr7	dehydrocholesterol reductase	NM_007856	3E-36	+
**C0701**	Scl27a1	solute carrier family 27A1	NM_198580	0.0	+
**C0901**	Acaa1	Acetyl-CoA acyltransferase 1	NM_130864	1E-77	+
**C4602**	Lpl	lipoprotein lipase	NM_008509	1E-37	-
**G0201**	Star	steroidogenic acute regulatory protein	NM_011485	7E-08	-
**6-Protein conformation or transport**					
**A3801**	Rpn1	ribophorin I	NM_133933	3E-14	-
**A4701**	Ergic3	ERGIC and golgi 3	NM_198398	6E-21	+
**A5302**	Gxylt1	glycosyltransferase 8 domain containing 3	NM_173601	0.0002	+
**C0601**	Ppia	peptidylprolyl isomerase A	NM_021130	7E-71	+
**C2001**	Grpel1	GrpE-like 1, mitochondrial	NM_024478	1E-13	+
**C2201**	Fxn	frataxin	NM_008044	4E-33	+
**C3602**	Slc26a9	solute carrier family 26, member 9	NM_177243	1E-9	+
**C7501**	Slc13a3	solute carrier family 13 member 3	NM_054055	3E-29	-
**G1801**	Slc15a1	Solute carrier family 15 member 1	NM_053079	0.0002	-
**G3702**	Rpn2	ribophorin II	NM_019642	3E-26	-
**G6101**	Nrbp1	nuclear receptor binding protein 1	NM_013392	4E-12	+
**G6601**	Slc6a8	solute carrier family 6 member 8	NM_005629	0.0001	-
**7-Cell cycle**					
**A0401**	Ingap	Mesocricetus auratus islet neogenesis associated protein	U41738	4E-79	-
**A3002**	Cdkn2b	cyclin-dependent kinase inhibitor 2B	NM_004936	1E-9	-
**A4801**	Ppp1cc	protein phosphatase 1, catalytic subunit, gamma	NM_002710	2E-78	+
**A5702**	Sec11a	SEC11 homolog A	NM_014300	1E-19	+
**C4002**	Mapk4	mitogen-activated protein kinase 4	NM_002747	9E-26	+
**C4701**	Ccndbp1	cyclin D-type binding-protein 1	NM_010761	1E-17	+
**C6301** **C6302**	Zw10	ZW10 homolog centromere/kinetochore protein	NM_004724	1E-04 1E-05	-
**8-Other functions**					
**A3903**	Rexo2	REX2, RNA exonuclease 2 homolog	NM_015523	6E-21	-
**A5403**	Hk2	hexokinase 2	NM_000189	4E-28	+
**A5602**	Pcbp2	poly(rC)-binding protein 2	NM_031989	1E-34	+
**A7301**	Lyrm4	LYR motif containing 4	NM_201358	0.0004	-
**C1801**	Itpripl2	inositol 1,4,5-triphosphate receptor Interacting protein-like 2	NM_001033380	4E-11	+
**C2401**	Tsn	translin	NM_011650	4E-10	+
**C4501**	Trip4	thyroid hormone receptor interactor 4	NM_016213	0.0006	-
**C7102**	Tbrg3	transforming growth factor beta regulated gene 3	NR_027799	0.001	+
**G0101**	Psme4	proteasome activator subunit 4	NM_014614	4E-87	+
**G0501**	Gatad2a	GATA zinc finger domain containing 2A	NM_001113346	8E-15	+
**G0901**	Hoxa10	Homeobox A10 (HOXA10)	NM_008263	1E-47	+
**G1101**	Iap	Syrian hamster intracisternal A particle	M10134	3E-73	+
**G2303**	Zc3h12c	zinc finger CCCH type containing 12C	NM_001162921	7E-9	-
**G6801**	Fst	follistatin	NM_006350	3E-42	-
**Ribosomal proteins**					
**A3703**	Rpl37a	ribosomal protein L37a	NM_000998	0.001	+
**A3901** **C3901**	Mrpl45	mitochondrial ribosomal protein L45	NM_025927	2E-10 6E-59	-
**A3902**	Rps21	ribosomal protein S21	NM_001024	7E-14	+
**A4001**	./.	mitochondrial 12S ribosomal RNA	X84390	0.0	+
**G2002**	Rpl27a	60S ribosomal protein L27a	NM_000990	5E-18	+
**G3504**	Rpl10	ribosomal protein L10	NR_026898	4E-132	+
**G3601** **G3602**	Rpl28	ribosomal protein L28	NM_009081	3E-22 8E-180	+
**G5701**	Rpl22	ribosomal protein L22	NM_000983	0.00009	+

The DEHP effect is identified by (+) for 2.0-fold over-expression, (++) for 10-fold over-expression, (-) for 2.0-fold under-expression and (--) for 10.0-fold under-expression for at least one dose of DEHP. The 37 Differential Display fragments showing no match after comparison with the RefSeq database were not listed in the table. Id (Identification Number) represents the internal reference of the sequence used before the identification of Differential Display sequence.

Transcription and signal transduction is another biological process targeted by DEHP treatment. We found 22 up-regulated genes, among which 3 were up-regulated more than 10-fold (*rab1b*, a Ras oncogen family member, Homeobox 1 and Forkhead P1). Heat- shock response related genes (*hsp90, hsp70 *and *hsph1*) and the genes involved in promoter methylation (*mettl5, mett5d1*) were up-regulated. On the other hand, 7 genes were down-regulated (CCAAT/enhancer binding protein delta, methyl-CpG binding domain protein 1, Map kinase 3, Protease subunit 5, SWI/SNF related actin dependent regulator of chromatin, ATP dependent helicase 4 and Forkhead A3).

Xenobiotic metabolism genes such as cytochromes and glutathione S-transferases were also found to be differentially expressed, indicating a mobilization of cellular defence and detoxication systems. An up-regulation of *cyp1b1 *and *cyp2e1 *was registered, whereas *cyp2f2 *was found to be down-regulated. Concerning GST, the Pi family was over-expressed while the Theta and Mu families were down-regulated.

Differential Display results confirmed the down-regulation of *c-myc *and showed down-regulation of p53. A down-regulation of pro-apoptotic genes (*casp8, topors*...) and an over-expression of anti-apoptotic genes (*bcl10, nfkb1, sh3kbp1*...) were also observed.

### Expression of genes involved in the regulation of the cytoskeleton by qPCR

**Figure 2:**
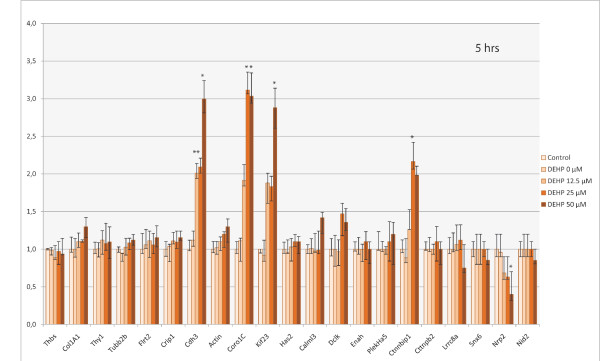
**Representative qPCR results of differentially-expressed genes involved in cytoskeleton regulation (according to the GO process), identified by Differential Display after 5 hrs of SHE cell exposure to DEHP**. These histograms show the ΔΔCt score normalized by gapdh mRNA level. Error bars represent the standard deviation of the ΔΔCt score. Only mRNA levels showing a two-fold increase or decrease at least, were considered indicative (*) of a change in gene expression.

**Figure 3:**
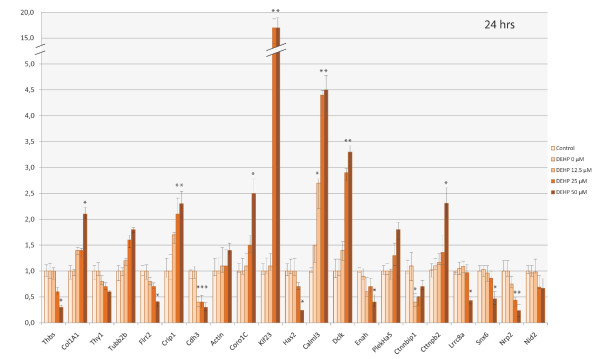
**Representative qPCR results of differentially-expressed genes involved in cytoskeleton regulation (according to the GO process), identified by Differential Display after 24 hrs of SHE cell exposure to DEHP**. These histograms show the ΔΔCt score normalized by gapdh mRNA level. Error bars represent the standard deviation of the ΔΔCt score. Only mRNA levels showing a two-fold increase or decrease at least, were considered indicative (*) of a change in gene expression.

The mRNA level of the 21 genes involved in the regulation of the cytoskeleton that were identified as differentially expressed after 24 hrs was confirmed by qPCR. The expression of these genes was also studied after exposure to DEHP for 5 hrs. Out of the 21 genes, four (*coro1C, kif23, cdh3 *and *cttnbip1*) were significantly up-regulated by DEHP treatment after 5 hrs of exposure (Figure 2) and one (*nrp2*) was significantly down-regulated. A clear dose-response relationship was observed for these 5 genes. After 24 hrs, these changes were confirmed for 3 genes (up-regulation of *coro1C *and *kif23*, and down-regulation of *nrp2*; Figure 3). Nevertheless, the down- and up-regulation was more pronounced after 24 hrs than after 5 hrs of DEHP exposure, for *nrp2 *and *kif23 *respectively. For instance, in cells exposed to 50 μM of DEHP, *Kif23 *was up-regulated 17-fold at 24 hrs versus 3-fold at 5 hrs. After 24 hrs, 5 other genes were significantly up-regulated (*col1A1, crip1, calml3, dclk*, and *cttnbp2*) by a factor ranging from 2.0 to 4.5 with a dose-related effect (Figure 3). Eight other genes were significantly down-regulated (*thbs1, flrt2, cdh3, has2, enah, ctnnbip1, lrrc8a *and *snx6*), with an expression ratio between 0.2 and 0.5 (corresponding to 5 and 2 fold down-regulation respectively). All these genes were down-regulated in a dose-dependent manner, except for *cdh3, enah, ctnnbip1, lrrc8a *and *snx6*. A threshold was observed with the latter genes (12.5 μM for *cdh3*; 50 μM for *enah, ctnnbip1, lrrc8a *and *snx6*). *Ctnnbip1 *was significantly down-regulated only for the lowest dose of DEHP (12.5 μM).

Although they had been identified as differentially expressed in DD, five genes (*thy1, tubb2b, β-actin, plekha5 *and *nid2*) were not shown to be significantly over- or under-expressed by qPCR. Yet the expression profiles of these genes indicated a dose-related increase for *tubb2b, β-actin *and *pleckha5 *but below the qPCR 2.0-fold threshold. As for *thy1 *and *nid2*, the dose-related decrease was inferior to 0.5.

### Expression of apoptosis-related genes, PPARs and CYP4 genes after DEHP treatment

**Figure 4:**
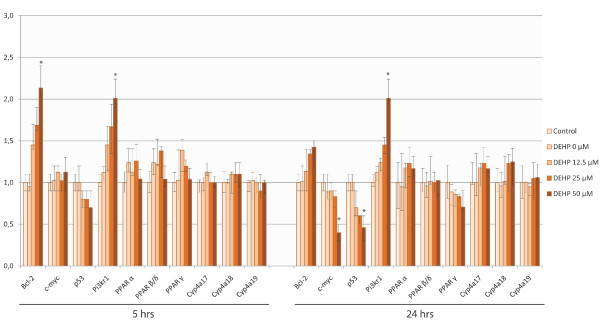
**Expression level of *bcl-2, c-myc, p53, pi3kr1*, PPARs and CYP4 family gene mRNA after treatment of SHE cells with DEHP, using qPCR**. These histograms show the ΔΔCt score normalized by the gapdh mRNA level. Error bars represent the standard deviation of the ΔΔCt score. Only mRNA levels showing a two-fold increase or decrease at least, were considered indicative (*) of a change in the gene expression. We observed a significant increase in the *bcl-2 *mRNA level after 5 hrs of exposure and a significant decrease in *c-myc *and *p53 *mRNA levels after 24 hrs of DEHP exposition. *Pi3kr1 *was found to be over-expressed for both lengths of exposure. None of PPARs or CYP4 genes was significantly over- or under-expressed after treatment.

The expression level of *bcl-2 *and *c-myc *mRNA was used as controls of DEHP effects. An increased level of *bcl-2 *after 5 hrs of exposure and a decreased level of *c-myc *after 24 hrs (Figure 4) were observed according to qPCR, as expected.

p53 was down-regulated in a dose- and time-dependent manner; a significant decrease of the mRNA level was found after 24 hrs at 50 μM DEHP.

None of the PPAR genes was identified as being differentially expressed by DD after DEHP exposure. In order to check these results, we measured the mRNA level of *PPARα, PPAR β/δ *and *PPAR γ*, by qPCR using hamster specific primers. No change in the expression of these genes was observed by qPCR after 5 or 24 hrs of exposure with DEHP in our study conditions (Figure 4). The same verification was carried out for CYP4 genes. Neither Differential Display nor qPCR allowed us to identify significant expression changes compared to the control.

## Discussion

The DDRT-PCR (Differential Display RT-PCR) technique was used in the present study to identify the differential mRNA expression patterns between control and DEHP-treated SHE cells. Indeed, this technique is still a method of choice for non-sequenced or partially-sequenced organisms and is able to identify nonabundant, rare or novel transcripts [36].

Using Differential Display, we found 122 genes whose expression was altered by DEHP treatment (79 up-regulated and 43 down-regulated). The concentrations studied (12.5, 25 and 50 μM) were in the range of concentrations that induced a morphological transformation of SHE cells, i.e. concentrations up to 77 μM for Mikalsen et al. [27] and in the range 25 μM-150 μM for Cruciani et al. [29].

We measured the mRNA level of genes involved in the regulation of the cytoskeleton using qPCR. This focus is justified by the fact that the modifications of cytoskeleton organization are early events in cell neoplastic process [37] and can be recorded in SHE cells after 7 days of exposure to carcinogenic agents in cell transformation assays. Morphological transformation affects a few percentage of the mixed population of SHE cells and all cell types [31]. From the present work, we can assume that the differentially-expressed genes measured in the first 24 hrs of exposure reflect the first targets of DEHP in the entire SHE cell population. The transcriptomic changes which were recorded correspond to the integrated mean of the cell responses significantly different in the exposed populations (p < 0.01), without consideration of cell specificity and sensitivity to DEHP. These significant expression changes in genes involved in cytoskeleton regulation, can be seen as early indicators of disturbances that will lead to cell transformation further in a few percentage of the most susceptible cells of the SHE population. The role of the cytoskeleton has been extensively studied in relation to invasion and metastasis, but little is known of its implication in the first stages of carcinogenesis. The identification of genomic changes associated with the triggering of cell transformation is useful from a mechanistic point of view and may be valuable in screening.

### Effects on cytoskeleton-related genes

DEHP was shown to affect several functions related to the cytoskeleton. The genes involved in cytoskeleton regulation and identified by Differential Display are listed in table 2. To summarize, DEHP affects actin polymerisation and stabilization, as well as cell-to-cell and cell-to-matrix adhesion processes. The expression of genes involved in organelle transport, in cytoskeleton remodelling, or adhesion in response to external factors was also modified by DEHP. These results are in line with the recent findings of Posnack et al. [38] who identified disturbances in mechanical adhesion function and protein trafficking in rats cardiomyocytes exposed to DEHP.

**Table 2:** List of genes involved in the regulation of the cytoskeleton and affected by DEHP

	5 hrs	24 hrs
**Up-regulated genes**	coro1C* (25 - 50 μM) kif23* (25 - 50 μM) cdh3* (12.5 - 25 - 50 μM) ctnnbip1* (25 μM)	coro1C* (25 - 50 μM) kif23* (25 - 50 μM) col1a1* (50 μM) crip1* (25 - 50 μM) calml3* (12.5 - 25 - 50 μM) dclk1* (25 - 50 μM) cttnbp2* (50 μM) *plekha5* *tubb2b* *β-actin*

**Down-regulated genes**	nrp2* (25 - 50 μM)	nrp2* (25 - 50 μM) thbs* (50 μM) flrt2* (50 μM) cdh3* (12.5 - 25 - 50 μM) has2* (50 μM) enah* (50 μM) lrcc8a* (50 μM) snx6* (50 μM) ctnnbip1* (12.5 μM) *nid2* *thy1*

This table summarizes the genes studied using qPCR. (*) indicates significant effects of DEHP (2.0-fold over- or under-expression) at concentration(s) specified in brackets. A trend for up- or down-regulation was found for the other genes (no concentration reported).

#### Actin polymerization and stabilization

To summarize the basic process, actin polymerization requires the Arp2/3 complex that needs to be stabilized by Enable Homolog (Enah) and is regulated by coronins. Enah is involved in the dynamic reorganization of the actin cytoskeleton, and stimulates nucleation and polymerization [39]. Coronins act on F-actin binding and bundling activities, but are able to inhibit the activity of Arp2/3 complex [40]. Actin polymers also require cortactin, which stabilizes nucleation sites for actin branching and elongation [41,42]. Crip1 facilitates actin filament bundling and stabilizes actin interaction with α-actinin too [43]. Linkage of actin polymers to adherens junctions, mainly composed of the transmembrane proteins cadherins, is insured through binding to α-catenin and β-catenin [44].

**Figure 5:**
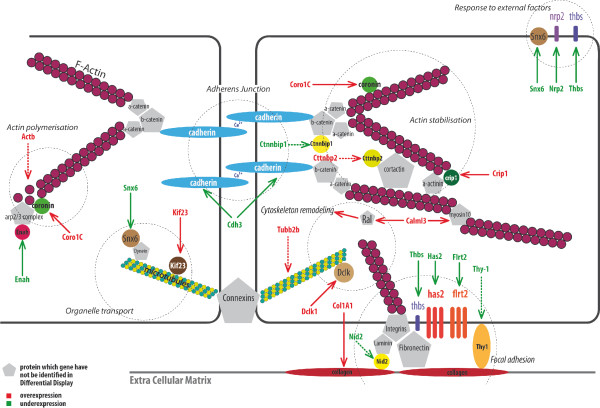
**Representative scheme of the genes affected by DEHP and involved in cytoskeleton regulation**. Genes in red have been found to be over-expressed using Differential Display (DD) and genes in green under-expressed. Dotted lines represent genes identified by DD but whose expression was not significant in qPCR.

Based on the gene expression data generated, we have tried to synthesize the effects of DEHP on actin organisation and cell adhesion specifically (Figure 5; over-expression in red; under-expression in green). A 5- and 24-hrs exposure to DEHP over-expressed Coronin 1C (Coro1C), resulting in F-actin disassembly [45]. Disorganization was amplified by under-expression of Enah involved in actin nucleation and polymerization, and expression of Cttnbp2 that counteracts cortactin which is known to stabilize the actin network. On the other hand, the binding of actin filaments to cadherins through catenin links appears to be reinforced owing to under-expression of Ctnnbip1 (a β-catenin blocker) and over-expression of Crip1, which intensifies fixation to actinin. Globally, the effects of DEHP on actin cytoskeleton disturb actin polymerization while intensifying binding on actinin and catenins. Posnack et al. [38] explored DEHP effects on rats cardiomyocytes in a range of concentrations two and three orders of magnitude higher than here. They found an over-expression of actinin, α-catenin and N-cadherin in a concentration-dependent manner.

#### Cell-cell and cell-matrix adhesion

Cell-cell adhesion and cell-matrix adhesion were also affected by DEHP treatment. The decrease in the P-Cadherin (Cdh3) mRNA level after 24 hrs of exposure indicates that DEHP weakened cell-cell contact, after a transient increase at 5 hrs of exposure for all doses tested. Weakening of cell-matrix adhesion may result from a decrease in the Hyaluronan synthase 2 (*has2*) mRNA level [46] and in Thrombospondin (Thbs1), an adhesive protein that interacts with fibronectin, laminin, integrins and collagen [47]. Loss of cell adhesion may also be explained by over-expression of Coro1C because this gene negatively regulates cell-matrix adhesion through focal adhesion kinase (FAK)-mediated signalling [45]. Also, under-expression of Enah, which is known to be involved in the control of cellular adhesion by the recruitment of proteins containing SH3- domain [48], contributes to the loss of cell-cell adhesion.

In addition, DEHP may lessen extracellular matrix adhesion by reducing the expression level of a number of transmembrane proteins involved in cell-matrix connections: Fibronectin leucine rich 2 (Flrt2) and Leucine rich repeat 8A (Lrrc8a) [49], Nidogen 2, which connects laminin-1 to the matrix [50], and Thy-1, which mediates fibroblastic adhesion [51] and is Thbs1 expression-dependent [52]. On the other hand, DEHP effects reinforce the extra-cellular matrix through an over-expression of *col1A1 *increasing collagen. This effect may be seen as a compensatory reaction to the weakening of cell-to-matrix link proteins by DEHP. Sobarzo et al. [53] demonstrated an up-regulation of N-cadherin and α-catenin in rat testis after 2 and 7 days of DEHP treatment, suggesting also a deregulation of cell adhesion molecules in seminiferous tubules.

DEHP decreases the response to external factors, such as the Vascular Endothelial Growth Factor (VEGF) or the Epidermal Growth Factor (EGF) through under-expression of neuropilin 2 (*nrp2*) and sorting nexin 6 (*snx6*) respectively. Nrp2 is a membrane receptor capable of binding VEGF and semaphorins, therefore its under-expression may inhibit cell adhesion and migration via the loss of integrins [54]. Snx6 is able to interact with EGF receptor and Transforming Growth Factor (TGF)-β receptor [55]. Under-expression of *snx6 *and *thbs1 *may lead to decreased interaction with Latent TGF Binding Protein (LTBP) in the upstream of the TGF-β pathway contributing to the repression of the TGF-β signaling pathway [56,57]. Under-expression of TGF-β is known to decrease apoptosis in rodent hepatocytes treated with peroxisome proliferators (PPs) [24].

#### Organelle transport and cytoskeleton remodelling

DEHP also interferes with functions of microtubules (composed of α- and β-tubulin). *Kif23*, which encodes a kinesin protein, was highly over-expressed after 5 hrs and 24 hrs of DEHP exposure. Kif23 has been shown to transport membranous organelles and protein complexes from cell nucleus to cell periphery in a microtubule- and ATP-dependent manner. Doublecortin-like kinase (Dclk) is a microtubule-associated protein encoding a Ca^2+^/calmodulin-dependent kinase. Its activities on binding and microtubule polymerization facilitate cell motility by remodelling the microtubule cytoskeleton [58]. Over-expression of *dclk *at 24 hrs of DEHP treatment is in line with an increased trend in β-tubulin (*tubb2b*).

Calmoduline-like 3 (*calml3*) was over-expressed after 24 hrs of DEHP exposure. Calmodulin (CaM) is a calcium-binding protein that translates the Ca^2+ ^signal into a wide variety of cellular processes, including the regulation of cytoskeleton remodelling acting with Caldesmon [59] or with Wnt pathway [60]. Calml3 is a CaM family member protein which increases cell motility by stabilizing and increasing myosin-10 for cell migration [61].

#### Other genes involved in signal transduction pathways and cytoskeleton regulation

We measured an over-expression level of phosphatidylinositol 3-kinase r1 (*pi3kr1*) using Differential Display and qPCR. Pi3k is a key signalling molecule in the PIP_3 _signalling transduction pathway and in actin reorganization and cell adhesion [62] and is able to regulate the synthesis of collagen I [63]. An activation of PI3K is also associated with a phosphorylation-dependent activation of Akt which contributes to tumorigenesis and metastasis [39]. The over-expression of *pi3kr1 *can be related to the under-expression of *ctnnbip1 *which interacts with β-catenin. In addition to the function of β-catenin in the actin cytoskeleton, its role in the regulation of Akt pathway activation [64] or in Wnt pathway regulation [65] is advanced. This protein forms part of a complex that captures growth and proliferation signals from the cell surface and is then activated to stimulate the expression of genes involved in cell proliferation. It would be worth studying β-catenin-dependent transcription in relation to carcinogenicity.

### DEHP effects in the SHE model compared to rats and mice

While the expression of *cyp1b1 and cyp2e1 *was up-regulated and *cyp2f2 *under-expressed, no change in expression level of CYP4 genes was found using DD and qPCR after DEHP exposure in our experimental conditions. CYP4 genes are said to be involved in peroxisome proliferation [66]. Eveillard et al. [67] who studied the involvement of DEHP in lipidogenesis in rats, found a slight increase in the PPARα level after 21 days of oral exposure to DEHP (200 mg/kg.day). They registered a significant increase in CYP4 levels after 14 days [68] and after 21 days [67] of exposure. On the other hand, we found no increased mRNA level of CYP4 and PPAR genes in DEHP-treated SHE cells. This underlines that the genes expression changes noted in the present study are independent of PPARs induction. Eveillard et al. [67] found that induced expression of *cyp2b10 *by DEHP was also independent of PPARα induction but CAR dependent. No change in CAR expression was registered in SHE cells, which may explain why no change in *cyp2b10 *was noted. Our results are consistent with the study of Ren et al. [20] who identified DEHP regulated genes independent of PPARα and CAR in rats and mice.

In our study, lipogenesis and xenobiotic metabolism pathways were impacted by DEHP, but not in a major prior way. This may be explained by the lower sensitivity of the hamster model compared with rats and mice to peroxisome proliferators [69]. Indeed, the Syrian hamster model presents an intermediate response between rats or mice and humans who are known to be non-responsive to PP induction [24]. The hamster model, like humans, is less responsive to PP induction than rats and mice, which is an advantage for mechanistic studies of PP effects and for screening human chemical carcinogens.

On the other hand, three genes (Actin β, Lipoprotein lipase, and Acetyl-Coenzyme A acyltransferase 1a) and 5 gene isoforms (Glutathione S-transferase-pi, -mu, -theta, CCAAT/enhancer binding protein and Nuclear receptor subfamily 1) were commonly found in our study and those carried out by Eveillard et al. [67,68], suggesting a pattern of response specific to DEHP.

Takashima et al. [70] also found similar responses in DEHP-treated mice. Up-regulation of *rab1b*, a RAS oncogene family member involved in cellular signal transduction or survival, was found in the latter study and in the present one. β-Tubulin was clearly over-expressed in mice, a trend which was noted in our study. Some gene isoforms of cadherin, nidogen, cyp1 family genes or LIM domain were also impacted in the liver of mice exposed to DEHP [70].

### DEHP effects on transcription factors

Other genes identified by Differential Display and involved in transcription and signal transduction pathways or apoptosis were also targeted by DEHP. A significant under-expression of *p53 *was found after 24 hrs of DEHP exposure using Differential Display and qPCR. This under-expression is in line with the anti- apoptotic effects of DEHP.

We confirmed the over-expression of *bcl-2 *after 5 hrs and the under-expression of *c-myc *after 24 hrs, events reported in a previous study on DEHP treated SHE cells in conditions similar to the present ones [26]. Map Kinases such as Mapk3, Mapk4 and Mapk15 were targeted by DEHP. Further investigations of Map Kinase pathways could be relevant due to their involvement in activities of transcription factors.

The G protein-coupled estrogen receptor (*gper*) was found to be over-expressed in Differential Display. Gper can be activated by estrogen-like compounds and its effect on cytoskeleton architecture has been reported [71]. Because of its implication in the regulation of MAPK [72] or TGF-β pathways [73], it would be worthwhile to investigate *gper *further.

### Performances of DD

The confirmation of differentially-expressed genes by qPCR showed that the expression levels of more than 75% of genes identified by DD were confirmed by qPCR. A comparative table of the sensitivity of DD versus qPCR is given in additional file 1. qPCR is more likely to quantify subtle changes in the expression level of mRNAs at different concentrations while DD seems to be more sensitive but is less discriminating. To summarize, 35% of the genes identified as differentially expressed in DD gave the same response at the same DEHP concentrations with qPCR while 40% were detected by DD at a lower DEHP concentration than with qPCR.

## Conclusion

Transcriptional responses of SHE cells to DEHP were studied in conditions inducing the cell neoplastic transformation, in order to identify gene expression changes in relation with effects of this non-genotoxic carcinogen. Functions impacted by DEHP were found to be PPAR-independent. Effects on cytoskeleton related genes indicated disturbances on actin polymerization and stabilization, cell-cell and cell-matrix adhesion and protein trafficking.

This is the first study that elucidates the genomic changes of DEHP on the organization of the cytoskeleton. Whether the expression changes of cytoskeleton-related genes identified here such as *coro1C, nrp2, kif23*, are specific to DEHP or to cell transforming agents more generally would require further studies. To answer, the gene sets identified as significantly over- or under-expressed in this study must be explored on other non-genotoxic carcinogens to identify biomarkers predictive of early events in the multistep carcinogenic process. Early disturbances in the expression of cytoskeleton-related genes should be considered good candidates.

## Methods

### Chemicals

DEHP (C.A.S. No. 117-81-7, purity 99%), purchased from Aldrich Chemicals (Gillingham, England) was dissolved in the DMSO solvent (C.A.S. No. 67.68.50). The latter was obtained from Sigma Aldrich (St Quentin Fallavier, France) and was used at a final concentration of 0.1%.

Nucleic acid stain Gelred purchased from Interchim (Montluçon, France) was used at a final concentration of 1:10000 (v/v).

All chemicals used for this study were electrophoresis grade or molecular biology grade. Their origin is specified in the following sections.

### SHE cell culture and treatment

SHE cells were isolated from Syrian hamster embryos at day 13 of gestation using the procedure described by Pienta et al. [74] and in accordance with the modifications suggested by Elias et al. [75]. Differentiated tissues, such as eyes, heart and viscera were removed and the remaining tissues were dissociated by dispase (1.2 U/ml). Stock cells were preserved in liquid nitrogen.

After thawing, passage-2 primary cells were pre-cultured until they reached 80% confluency. The culture medium was Dulbecco's modified Eagle medium (DMEM) (Gibco, Invitrogen, Cergy Pontoise, France) supplemented with 10% foetal calf serum (FCS) (Hyclone, Brebières, France; lot N°#ASB28835), 1.5 g/L NaHCO_3 _at 37°C in a 10% CO_2 _humidified atmosphere and pH 7.0. No phenol red was added to the medium.

Cells used for the DEHP studies were sampled from a monolayer during the growing phase, 48 hrs after seeding. Cells were trypsinized and treated during replating with DEHP at concentrations of 0 μM (vehicle control), 12.5 μM, 25 μM and 50 μM in DMEM culture medium supplemented with 10% FCS. Cells were then incubated for 5 hrs and 24 hrs at 37°C in a 10% CO_2 _humidified atmosphere.

### RNA isolation

Total RNA extractions were performed directly in the dish, using Nucleospin RNA II Extract Kit (Macherey Nagel, Hoerdt, France), according to the manufacturer's instructions. A DNAse I treatment was performed directly through the column used to collect RNAs and before the elution phase of DNA-free RNA.

RNA was quantified by spectrophotometry (Nanodrop, Labtech) measuring the A_260_/A_280 _ratio and its quality was ensured by electrophoresis using a 1% RNase-free agarose gel. Aliquots were stored at -80°C before use for Differential Display and Real-time PCR.

### Anchored Reverse transcription (RT) and Differential Display

The Differential Display was performed as described by Liang et al. [35], with minor modifications concerning DD fragment revelation with GelRed.

For Differential Display, three separate RT reactions were performed with a different one-base anchored oligo-dT primer (H-dTA, H-dTC and H-dTG) to produce three different subsets of cDNA pools. The sequences of the anchored and the arbitrary primers are given as additional file 2. The RT reactions were carried out using 2 μL of each primer (50 μM) and 4 μg of total RNA. 8 μL of RevertAid M-MuLV Reverse Transcriptase 5x reaction buffer (Fermentas, Saint-Rémy-lès-Chevreuse, France), 1.5 μL of 10 mM dNTPs (Fermentas, Saint-Rémy-lès-Chevreuse, France) and up to 35 μL Nuclease-free water were added to each tube, mixed, then heated at 70°C for 3 min. Tubes were centrifuged and incubated on ice for 5 min, then 2 μL (40 U) of RNaseOUT Recombinant RNase Inhibitor (Invitrogen, Cergy Pontoise, France), 1 μL (200 U) of RevertAid M-MuLV RT (Fermentas, Saint-Rémy-lès-Chevreuse, France) and 2 μL of Nuclease-free water were added to each tube. Each tube was mixed by gentle pipetting then incubated in a thermocycler at 42°C for 1 h, followed by 95°C for 10 min. The tubes were then centrifuged and stored at -80°C until use.

Amplification was then performed using combinations of the three original anchored primers from the reverse transcription step and eighty arbitrary 13-mers (H-AP), giving a total of 240 amplification combinations. All reactions contained 2 μL of a 10x PCR buffer containing 25 mM of MgCl_2_, 1.6 μL of 1 mM dNTP mix, 1 U of Taq Polymerase (Euromedex, Souffelweyersheim, France) and the primer combination at a final concentration of 1 μM. Tubes were incubated for 5 min at 95°C. The next 40 cycles were 95°C for 30 s (denaturation), 40°C for 2 min (annealing), 72°C for 1 min (amplification). A final extension of 72°C for 10 min completed the cycle.

After thermocycling, PCR-amplified fragments were resolved in a 6% native polyacrylamide gel in 1 × TBE buffer (89 mM Tris base/89 mM boric acid/2 mM EDTA, pH 8.0), using 10 μL of PCR product mixed with 2 μl of loading buffer (0.05% xylene cyanol, 40% sucrose, 20 mM EDTA, pH 8.0). Gels were run at 100 V for 20 hrs, then the fragments were stained with GelRed 1X in water, for 30 min in the dark. Bands on the gel were revealed on a UV-transluminator. PCR products that showed differential expression between control and treated samples were identified with QuantityOne^® ^1-D analysis software (BioRad, Marne-la-Coquette, France).

Bands which were up- or down- regulated more than 2-fold, were selected and characterized in the next step of analysis. Differentially-expressed bands were excised, reamplified and their sizes were checked before cloning. To summarize, fragments of interest were recovered using a clean razor blade and extracted from the gel matrix by boiling in 200 μL of buffer (10 mM Tris/1 mM EDTA/1% SDS (w/v), pH 8.0) for 15 min. After overnight precipitation at -80°C, the eluted DNA was reamplified using the same primers and PCR conditions as the ones used in the DD-PCR step. Reamplified DNA was run in a 1.5% agarose gel containing 1X GelRed and recovered using NucleoSpin ^® ^Extract II kit (Macherey Nagel, Hoerdt, France) before cloning.

Cloning was carried out using a TA Cloning Kit (pGEM-T, Promega, Charbonnières, France), according to the manufacturer's instructions. Plasmid DNA was extracted from the cultures using Nucleospin ^® ^Plasmid QuickPure (Macherey Nagel, Hoerdt, France), according to the manufacturer's instructions and sequenced bidirectionally by the DNA sequencing service of MWG Operon (Ebersberg, Germany), using T7 and SP6 primers.

### Identification of differentially-expressed genes

Sequences were compared with the National Centre of Biotechnology Information Gene Bank database (http://www.ncbi.nlm.nih.gov) using the tBLASTx algorithm and RefSeq mouse or Refseq human as a reference.

### Confirmation of differentially-expressed sequences by Quantitative Real Time PCR (qPCR)

First-strand cDNA was synthesized from 2 μg of total RNA using VILO-Superscript™ III reverse transcriptase (Invitrogen, Cergy Pontoise, France) and random-hexamer primers. To summarize, 2 μg of total RNA was combined with 4 μL of 5X VILO™ reaction mix (containing RT buffer, MgCl_2_, dNTPs and random primers) and 2 μL of 10X enzyme mix (containing Superscript^® ^III and RNase inhinitor). The final volume was adjusted to 20 μL and the reaction mix was incubated at 42°C for 60 min. Then, cDNAs were diluted 20-fold, according to the manufacturer's instructions, before qPCR amplifications. The oligonucleotides used as primers in the quantitative real time PCR assay are described in table 3. If possible, at least one primer in each pair spanned an exon-intron boundary. PCR was carried out using Fast SYBR^®^Green Master Mix (Applied Biosystem, Courtaboeuf, France). Amplifications were performed on a StepOnePlus Real-Time PCR system (Applied Biosystem, Courtaboeuf, France). Each qPCR reaction contained 10 μL of 2X Fast SYBR^®^Green Master Mix, 5 μL of primers, 2 μL of diluted cDNA and 3 μL of Nuclease-free water. Amplification parameters were set as follows: initial denaturation (95°C, 3 min), and then amplification (95°C, 3 s and 60°C 30s) for 40 cycles. Glyceraldehyde 3-phosphate dehydrogenase (*gapdh*) mRNA level was used as a housekeeping gene to normalize qPCR data. This gene was chosen because DEHP exposure did not affect its expression unlike *β-actin *which was also tested (data not shown). qPCR results were analyzed using the software provided with the thermocycler and DataAssist, using the ΔΔCt method [76]. Each validated primer pair used yielded a single peak of dissociation on the melting curve. The efficiency calculated by standard curve with five log-10 dilution points was between 0.95 and 1.05. A 2.0-fold threshold and a p-value of 0.05 were used to determine the significance of differential expression levels according to the standard parameters of DataAssist.

**Table 3:** List of the primers used for real-time qPCR

	Genes	Accession N°	Primers sequence (5'-3')
**Housekeeping gene**			
***gapdh***	Glyceraldehyde 3-phosphate dehydrogenase	DQ403055	F: CAATGACCCCTTCATTGACC R: GACAAGCTTCCCGTTCTCAG
**Regulation of cytoskeleton**			
***tubb2b***	β-Tubulin	NM_023716	F: GCAACATGAATGACCTGGTG R: ACCAGAGACCCAGCACAAAC
***thy1***	Thy1 Antigen	NM_009382.3	F: AAGGCCTCTGCCTGTAGTGA R: GAAGAGGCAGGTTGCAAGAC
***actin***	β-Actin	NM_007393	F: CACCACCACAGCCGAGAG R: CCAGGGAGGAAGAGGATGC
***col1a1***	Collagen α1	NM_000088	F: GGGTCATTTCCACATGCTTT R: TCCGGGTTTCAGAGTACCAC
***thbs1***	Thrombospondin 1	NM_011580	F: CCAAAGCCTGCAAGAAAGAC R: CCTGCTTGTTGCAAACTTGA
***plekha5***	Plekstrin homology A5	NM_019012	F: GTGCATCTGCCTGAAGACAA R: TGGGAACCTTTAACGACTGG
***kif23***	Kinesin 23	NM_024245.4	F: CCTGAGCTTTCCTGACCAAG R: AGTTCCTTCTGGGTGGTGTG
***has2***	Hyaluronan synthase 2	NM_008216	F: CGGAGGACGAGTCTATGAGC R: TTTTCCGGTGTTCCAAAAAG
***flrt2***	Fibronectin leucine rich 2	NM_201518	F: ACCGCACTGTGGAAGATACC R: GCAAGACAACGAGCACAAAA
***enah***	Enabled homolog	NM_008680	F: GCCTATGCTTCAGCACTTCC R: GGGCGATTGTCTTCTGACAT
***dclk1***	Doublecortin like 1	NM_019978	F: AGCCTCCACCAGCTCAGTTA R: CCATACACATCGCTCCATTG
***ctnnbip1***	Catenin β interacting protein 1	NM_020248	F: TTGGCTGCAGAAAGAAACCT R: CCAGCCAATCACAACCTTTT
***crip1***	Cystein rich protein	NM_007763	F: AGTCCAGAGCCTGCAACCTA R: GGAGTAGCAGGGATGATTGC
***coro1c***	Coronin	NM_014325	F: GCAGAAGAGTGGTTCGAAGG R: TGATCAGGTCGCACTTCTTG
***cdh3***	Cadherin 3	NM_007665	F: CACACGACCTCATGTTCACC R: CTGTACCTCATGGCCCACTT
***calml3***	Calmodulin like 3	NM_027416.3	F: ATCGACAAGGATGGAAACG R: ATCTACCTCCTCGTCGCTCA
***cttnbp2***	Cortactin binding protein 2	NM_030249	F: GACAAAGAAGGCTGGACTGC R: CTCACCCACGGAAATCCTTA
***lrrc8a***	Leucine rich repeat 8A	NM_177725	F: AGAGCCCACTTACCCCAACT R: GTGTGCAGAAGCACGAGGTA
***snx6***	Sorting nexin 6	NM_021249	F: CCAAGACCTGATTTTGATGCTTC R: CATGCATCGCAACTGTCTTC
***nrp2***	Neuropilin 2	NM_0010774	F: ATAAGCACTGATGTCCCACTG R: GAGTTGCTCCAATCTCCTTCA
***nid2***	Nidogen 2	NM_008695	F: CTCATTCAGTTGTGCCTGC R: ATAGCTGCCTCATGACATCG
**Regulation of apoptosis**			
***pi3kr1***	phosphatidylinositol 3-kinase (p85a)	NM_001077495	F: CGAGCCCGACCGGAGGTGAA R: CGCACACTGCCGTCCGAGTT
***bcl-2***	B-cell lymphoma 2	AJ582074	F: CGCAGAGATGTCCAGTCAGC R: CGAACTCAAAGAAGACCACAA
***c-myc***	cellular myelocytomatosis oncogene	AJ582076	F: GACCCTGATTCGGACCTCTT R: CGACTCCACAGCCTTCTCTC
***p53***	tumor supressor p53	U07182	F: ATGACGGAAGTTGTAAGAC R: TCGGATAAGATGCTGAGG
**PPARs genes**			
***ppar α***	peroxisome proliferator activated receptor alpha	AY170844	F: GTTTCTTTCGGCGAACTATT R: ACACGTGAGAATCTCTGCTT
***ppar β/δ***	peroxisome proliferator activated receptor beta/delta	AF486582	F: TGCAAGATCCAGAAGAAGAA R: GTAGATGTGCTTGGAGAAGG
***ppar γ***	peroxisome proliferator activated receptor gamma	AB525757	F: GGACCTCTCTATGATGGATG R: GGATGCAGGTTCTACTTTGA
**CYP4 family genes**			
***cyp4a17***	Cytochrome P450 4A17	AJ555628	F: ACCAGATGCCCTACACTACC R: GTGCGTAAATGGAGAGTACA
***cyp4a18***	Cytochrome P450 4A18	AJ555629	F: ACCAGATGCCCTACACTACC R: GGCCATAAATGGAGATTGCA
***cyp4a19***	Cytochrome P450 4A19	AJ555630	F: ACCAGATGCCCTACACTACC R: GTGCATAAATGGAGAGTGTG

## Authors' contributions

YL performed Differential Display, carried out qPCR analysis and was involved in the design of experiment. PP and FA were involved in the design of the experiment study and technical assistance. PV involved in the overall design and coordination of the study. All authors participated in the writing of the manuscript and approved it.

## Supplementary Material

Additional file 1 **Comparative table of the sensitivity of DD versus qPCR**. Comparisons between the ratio of bands intensity compared to the control on the gel using an image analysis software (QuantityOne^® ^1-D analysis software BioRad, Marne-la-Coquette, France) for Differential Display and the ΔΔCt score normalized by gapdh mRNA level after analysis with StepOne and DataAssist (Roche Applied Biosystem, Courtaboeuf, France) for qPCR. Click here for file

Additional file 2 **Primers for Differential Display**. List of the sequences of the anchored and the arbitrary primers used for the Differential Display experiments. Click here for file
