# Supplementary material for: Transcriptomic effects of di-(2-ethylhexyl)-phthalate in Syrian hamster embryo cells: an important role of early cytoskeleton disturbances in carcinogenesis?
Source: BMC Genomics. 2011 Oct 25;12:524. doi: 10.1186/1471-2164-12-524 (PMC3218109; doi:10.1186/1471-2164-12-524)
Supplement: Additional file 1 — Comparative table of the sensitivity of DD versus qPCR. Comparisons between the ratio of bands intensity compared to the control on the gel using an image analysis software (QuantityOne® 1-D analysis software BioRad, Marne-la-Coquette, France) for Differential Display and the ΔΔCt score normalized by gapdh mRNA level after analysis with StepOne and DataAssist (Roche Applied Biosystem, Courtaboeuf, France) for qPCR. [file 1471-2164-12-524-S1.PDF]

Anchored olio-dT primers

dT+A : AAGCTTTTTTTTTTTTTA
dT+ C : AAGCTTTTTTTTTTTTTC
dT+ G : AAGCTTTTTTTTTTTTTG

Arbitrary Primers (H-AP1 to H-AP80)

H-AP1 :	AAGCTTGATTGCC	H-AP41 :	AAGCTTACGGGGT
H-AP2 :	AAGCTTCGACTGT	H-AP42 :	AAGCTTTGCACCG
H-AP3 :	AAGCTTTGGTCAG	H-AP43 :	AAGCTTGAAGCGG
H-AP4 :	AAGCTTCTCAACG	H-AP44 :	AAGCTTCTCCGGA
H-AP5 :	AAGCTTAGTAGGC	H-AP45 :	AAGCTTGGCTGAC
H-AP6 :	AAGCTTGCACCAT	H-AP46 :	AAGCTTCGGTCCT
H-AP7 :	AAGCTTAACGAGG	H-AP47 :	AAGCTTATGCCCG
H-AP8 :	AAGCTTTTACCGC	H-AP48 :	AAGCTTGCGGTGA
H-AP9 :	AAGCTTCATTCCG	H-AP49 :	AAGCTTTAGTCCA
H-AP10 :	AAGCTTCCACGTA	H-AP50 :	AAGCTTTGAGACT
H-AP11 :	AAGCTTCGGGTAA	H-AP51 :	AAGCTTCGAAATG
H-AP12 :	AAGCTTGAGTGCT	H-AP52 :	AAGCTTGACCTTT
H-AP13 :	AAGCTTCGGCATA	H-AP53 :	AAGCTTCCTCTAT
H-AP14 :	AAGCTTGGAGCTT	H-AP54 :	AAGCTTTTGAGGT
H-AP15 :	AAGCTTACGCAAC	H-AP55 :	AAGCTTACGTTAG
H-AP16 :	AAGCTTTAGAGCG	H-AP56 :	AAGCTTATGAAGG
H-AP17 :	AAGCTTACCAGGT	H-AP57 :	AAGCTTGTTGGTA
H-AP18 :	AAGCTTAGAGGCA	H-AP58 :	AAGCTTAACTGAG
H-AP19 :	AAGCTTATCGCTC	H-AP59 :	AAGCTTCTAGCAT
H-AP20 :	AAGCTTGTTGTGC	H-AP60 :	AAGCTTTCGAATC
H-AP21 :	AAGCTTTCTCTGG	H-AP61 :	AAGCTTAGTTGCT
H-AP22 :	AAGCTTTTGATCC	H-AP62 :	AAGCTTGCAAGTT
H-AP23 :	AAGCTTGGCTATG	H-AP63 :	AAGCTTTTATCCG
H-AP24 :	AAGCTTCACTAGC	H-AP64 :	AAGCTTCATATGC
H-AP25 :	AAGCTTTCCTGGA	H-AP65 :	AAGCTTCAAGACC
H-AP26 :	AAGCTTGCCATGG	H-AP66 :	AAGCTTGCCTTTA
H-AP27 :	AAGCTTCTGCTGG	H-AP67 :	AAGCTTTATTTAT
H-AP28 :	AAGCTTACGATGC	H-AP68 :	AAGCTTCTTTGGT
H-AP29 :	AAGCTTACGAGCA	H-AP69 :	AAGCTTAATAACG
H-AP30 :	AAGCTTCGATCGT	H-AP70 :	AAGCTTTCATATG
H-AP31 :	AAGCTTGGTGAAC	H-AP71 :	AAGCTTGTAGTAA
H-AP32 :	AAGCTTCTTGCAA	H-AP72 :	AAGCTTTCAAAGA
H-AP33 :	AAGCTTGCTGCTC	H-AP73 :	AAGCTTAGTTATC
H-AP34 :	AAGCTTCAGCAGC	H-AP74 :	AAGCTTCAAGTTT
H-AP35 :	AAGCTTCAGGGCA	H-AP75 :	AAGCTTTTATTCG
H-AP36 :	AAGCTTCGACGCT	H-AP76 :	AAGCTTGTTATAG
H-AP37 :	AAGCTTGGGCCTA	H-AP77 :	AAGCTTTGAATTC
H-AP38 :	AAGCTTCCAGTGC	H-AP78 :	AAGCTTAAATCGA
H-AP39 :	AAGCTTTTCGCAG	H-AP79 :	AAGCTTGTCTAAA
H-AP40 :	AAGCTTGTCAGCC	H-AP80 :	AAGCTTCTATTTC

S2 : Sequences of the anchored primers and the arbitrary primers used for Differential Display.
