# Supplementary material for: Transcriptomic effects of di-(2-ethylhexyl)-phthalate in Syrian hamster embryo cells: an important role of early cytoskeleton disturbances in carcinogenesis?
Source: BMC Genomics. 2011 Oct 25;12:524. doi: 10.1186/1471-2164-12-524 (PMC3218109; doi:10.1186/1471-2164-12-524)
Supplement: Additional file 2 — Primers for Differential Display. List of the sequences of the anchored and the arbitrary primers used for the Differential Display experiments. [file 1471-2164-12-524-S2.PDF]

		24 HOURS				
		Control	DEHP 0µM	DEHP 12.5 µM	DEHP 25µM	DEHP 50 µM
Thbs1	DD		=	=	-	-
	qPCR	1.0	1.0	1.0	0.6	**0.3**
Col1a1	DD		=	+	+	+
	qPCR	1.0	1.0	1.4	1.4	**2.1**
Thy1	DD		=	=	=	-
	qPCR	1.0	1.0	0.8	0.7	0.6
Tub2b	DD		=	=	+	+
	qPCR	1.0	1.0	1.2	1.6	1.8
Flrt2	DD		=	=	=	-
	qPCR	1.0	1.0	0.8	0.7	**0.4**
Crip1	DD		=	+	+	+
	qPCR	1.0	1.0	1.7	**2.1**	**2.3**
Cdh3	DD		=	=	-	-
	qPCR	1.0	1.0	**0.4**	**0.4**	**0.3**
Actin	DD		=	=	=	+
	qPCR	1.0	1.0	1.1	1.1	1.4
Coro1C	DD		=	=	=	+
	qPCR	1.0	1.0	1.1	1.5	**2.5**
Kif23	DD		=	++	++	++
	qPCR	1.0	1.0	1.1	**16.4**	**16.6**
Has2	DD		=	=	=	-
	qPCR	1.0	1.0	1.0	0.7	**0.2**
Calml3	DD		=	+	+	+
	qPCR	1.0	1.0	**2.7**	**4.4**	**4.5**
Dclk	DD		=	+	+	+
	qPCR	1.0	1.0	1.4	**2.9**	**3.3**
Enah	DD		=	=	=	-
	qPCR	1.0	0.9	0.6	0.7	**0.4**
PlekHa5	DD		=	=	=	+
	qPCR	1.0	1.0	1.0	1.3	1.8
Ctnnbip1	DD		=	-	-	-
	qPCR	1.0	1.1	**0.4**	**0.5**	0.7
Cttnbp2	DD		=	=	+	+
	qPCR	1.0	1.1	1.2	1.4	**2.3**
Lrrc8A	DD		=	=	=	-
	qPCR	1.0	1.1	1.1	1.0	**0.4**
Snx6	DD		=	=	=	-
	qPCR	1.0	1.0	1.0	0.9	**0.5**
Nrp2	DD		=	-	-	-
	qPCR	1.0	1.0	0.8	**0.4**	**0.2**
Nid2	DD		=	=	=	-
	qPCR	1.0	1.0	1.0	0.7	0.7

S3: The DEHP effect is identified by (+) for 2.0-fold overexpression, (++) for 10-fold overexpression, (-) for 2.0-fold underexpression and (--) for 10.0-fold underexpression for Differential Display and corresponds to the ratio of bands intensity compared to the control on the gel using an image analysis software (QuantityOne® 1-D analysis software BioRad, Marne-la-Coquette, France). For qPCR, the values represent the ΔΔCt score normalized by gapdh mRNA level after analysis with StepOne and DataAssist (Roche Applied Biosystem,; Courtaboeuf, France). This table showed that 75% of genes identified by DD are confirmed in qPCR.
